# Alcohol use disorders and associated chronic disease – a national retrospective cohort study from France

**DOI:** 10.1186/s12889-017-4587-y

**Published:** 2017-07-21

**Authors:** Michaël Schwarzinger, Sophie Pascale Thiébaut, Sylvain Baillot, Vincent Mallet, Jürgen Rehm

**Affiliations:** 1 Translational Health Economics Network (THEN), 39 quai de Valmy, 75010 Paris, France; 2 Institut Cochin, Université Paris Descartes, Sorbonne Paris Cité, (UMR-S1016), CNRS (UMR 8104), Paris, France; 3 Institut Pasteur, Centre d’Immunologie Humaine (UMS20), Paris, France; Hepatology, Assistance Publique - Hôpitaux de Paris, Groupe Hospitalier Cochin Port-Royal, Paris, France; 4 Institute for Mental Health Policy Research, Centre for Addiction and Mental Health, 33 Russell Street, Toronto, ON M5S 2S1 Canada; 5 Campbell Family Mental Health Research Institute, Centre for Addiction and Mental Health, 33 Russell Street, Toronto, ON M5S 2S1 Canada; 6 Addiction Policy, Dalla Lana School of Public Health, University of Toronto, 155 College Street, 6th floor, Toronto, ON M5T 3M7 Canada; 7 Institute of Medical Science, University of Toronto, Faculty of Medicine, Medical Sciences Building, 1 King’s College Circle, Room 2374, Toronto, ON M5S 1A8 Canada; 8 Department of Psychiatry, University of Toronto, 250 College Street, 8th floor, Toronto, ON M5T 1R8 Canada; 9 Institute of Clinical Psychology and Psychotherapy & Center of Clinical Epidemiology and Longitudinal Studies (CELOS), Technische Universität Dresden, Chemnitzer Str. 46, 01187 Dresden, Germany

**Keywords:** Alcohol use disorders, Risk factor, Mortality, Non-communicable disease, Burden of disease

## Abstract

**Background:**

Evidence on diseases caused by or associated with alcohol use disorders (AUDs) has been based on two meta-analyses including rather dated studies. The objective of this contribution was to estimate the risks of all-cause mortality and alcohol-attributable disease categories depending on a diagnosis of AUDs in a national sample for France.

**Methods:**

In a national retrospective cohort study on all inpatient acute and rehabilitation care patients in Metropolitan France 2008–2012 (*N* = 26,356,361), AUDs and other disease categories were identified from all discharge diagnoses according to standard definitions, and we relied on in-hospital death for mortality (57.4% of all deaths).

**Results:**

704,803 (2.7%) patients identified with AUDs had a threefold higher risk of death (HR = 2.98; 95% CI: 2.96–3.00) and died on average 12.2 years younger (men: 10.4, 95% CI: 10.3–10.5; women: 13.7, 95% CI: 13.6–13.9). AUDs were associated with significantly higher risks of hospital admission for all alcohol-attributable disease categories: digestive diseases, cancers (exception: breast cancer), cardiovascular diseases, dementia, infectious diseases, and injuries. Elevated risks were highest for liver diseases that were associated with about two-third of deaths in patients with AUDs (men: 64.3%; women: 71.1%).

**Conclusions:**

AUDs were associated with marked premature mortality and higher risks of alcohol-attributable disease categories. Our results support the urgent need of measures to reduce the burden of AUDs.

**Electronic supplementary material:**

The online version of this article (doi:10.1186/s12889-017-4587-y) contains supplementary material, which is available to authorized users.

## Introduction

### Background/rationale

Alcohol use is a major risk factor for burden of disease and injury [1, 2]. Alcohol use disorders (AUDs), defined as alcohol dependence and harmful use of alcohol (cf. International Classification of Disease tenth revision (ICD-10) [3]), contribute markedly to this burden [4, 5]. In the European Union, in 2004, alcohol dependence, the most severe disabling of AUDs, was estimated to account for 62% of all burden from alcohol use [4].

This contribution aims to quantify the hospitalization and mortality risks associated with AUD for all disease categories are causally related to alcohol, i.e., assuming level of ethanol exposure as the pathway between AUD and outcomes (general: [6]; for risk analyses of ethanol e.g.: [7, 8]). Under this assumption, all conditions causally impacted by either chronic or episodic heavy alcohol use [9–11] would be relevant: all alcohol-attributable cancers [12]; liver cirrhosis [9], acute and chronic pancreatitis [13] as digestive diseases; ischemic heart disease [14], congestive heart disease [15], atrial fibrillation [16], stroke [17], as cardiovascular diseases; and pneumonia [18] and tuberculosis [19] as infectious diseases; and both intentional and unintentional injury [20]. Mental disorders are not on this list, as causality is not clear and quantification of the causal impact of alcohol or alcohol use disorders has not yet been achieved [21]. Alzheimer’s disease and other forms of dementia may be an exception there. On the one hand, there is a possible protective effect of light to moderate drinking [22, 23]. On the other hand, several subtypes of dementia are clearly detrimentally and causally related to heavy drinking [24], and the most recent review exhibited a J- or U-shaped relationship between the intensity of alcohol consumption and the sign of the effect [25]. We included dementia in our list for quantification, even though in prior comparative risk analyses for alcohol, this condition was excluded [1, 26, 27].

Two meta-analyses of cause-specific mortality risks of AUDs corroborated the above list [28, 29]. Two points related to these two meta-analyses deserve to be mentioned. Firstly, most of the evidence on diseases caused or associated with AUDs was rather old, and secondly, significant heterogeneity was found between study results.

### Objective

Based on this background, the main objective was to estimate the hospitalization and mortality risks of all-cause mortality and alcohol-attributable disease categories depending on a diagnosis of AUDs in a national sample for France.

## Methods

### Data source

Main data source was the French National Hospital Discharge database (Programme de Médicalisation des Systèmes d’Information) which contains all public and private claims for inpatient acute and rehabilitation care since 2008 [30]. The standardized discharge summary includes: patient’s demographics; primary and associated discharge diagnosis codes according to WHO International Classification of Diseases, tenth revision (ICD-10, French version); medical procedures performed (French Medical Common Procedure Coding System); length of stay, entry and discharge modes (including in-hospital death). The French National Hospital Discharge database was analysed with de-identified patient information for the years 2008 to 2012. The unique anonymous identifier allows linking all hospital claims of the patient and tracking the occurrence and progression of severe conditions over time.

### Study population

Out of 28,953,755 adults residing in Metropolitan France with at least one hospital record in the 2008–2012 National Hospital Discharge database, we excluded 2,597,394 (9.0%) women only admitted for delivery care or abortion during the study period.

### Alcohol use disorders

Patients with AUDs were identified by any hospital record describing mental and behavioural states due to former or current harmful use of alcohol (ICD-10 F10.1 to F10.9) or ICD-10 codes indicating a disease onset that is fully attributable to AUDs (e.g., K70 ‘Alcoholic Liver Disease’). Individuals identified with AUDs were further categorized as ‘abstinent’ if they had any hospital record of abstinence in the years 2008–2012 (see Additional file 1: Table S1 for ICD-10 codes used).

### Alcohol-attributable disease categories

We investigated risk relationships between AUDs and alcohol-attributable disease categories requiring hospital care: 1) liver diseases (cirrhosis and liver cancer); 2) pancreatic diseases (acute and chronic pancreatitis, pancreatic cancer); 3) upper aerodigestive tract cancer (oral cavity, oropharynx, hypopharynx, larynx, and oesophageal cancers); 4) other cancers (colorectal or breast cancers); 5) cardiovascular diseases (ischemic heart diseases, congestive heart failure, atrial fibrillation, and cerebrovascular diseases disentangling ischemic or haemorrhagic stroke); 6) dementia; 7) infectious diseases (pneumococcal pneumonia, and tuberculosis); and 8) injuries (non-intentional injury, self-harm, and other intentional injury). Disease categories were defined by the presence of ICD-10 codes as primary or associated discharge diagnosis, and ICD-10 codes were selected to concur with the definitions used in GLOBOCAN (http://globocan.iarc.fr/Default.aspx), the Global Burden of Disease study, or the Charlson Comorbidity Index (see Additional file 1: Table S1 for ICD-10 codes used).

### Statistical analysis

All-cause mortality risk and cause-specific risks of hospital admission were estimated in Cox proportional hazards models with left truncation and right censoring. For all-cause mortality risk, we used age as the time-scale to estimate the effect of AUDs over lifetime with universal follow-up of the French adult population from January 1st, 2008, until death or last discharge in 2008–2012 [31]. For each cause-specific risk of hospital admission, we used similarly age as the time-scale from January 1st, 2008, until first hospital record of the disease category, death, or last discharge in 2008–2012. All Cox models were stratified on gender, five main French regions (North-West, North-East, Greater Paris area, South-West, South-East), and having received care in a tertiary care center (public teaching hospital or comprehensive cancer care centre) since Schoenfeld residuals tests pointed out non proportionality of the effects for these variables.

The effect of AUDs over lifetime was suspected to be non-proportional as AUDs are likely to have different effects at different ages, either because of the frailty of patients with AUDs or because age indicates the duration of exposure to alcohol. We examined hazard ratios under two assumptions: (i) one binary variable of AUDs status was introduced under the assumption of linearity of the effect of AUDs over lifetime; (ii) an age-varying effect of AUDs was estimated by introducing a third order polynomial of age. All analyses were performed with Statistical Analysis System (SAS 9.4).

### Potential sources of bias for mortality calculations

While most alcohol-attributable disease categories require hospital care and thus were covered by our national hospital data sources, the main concern about the validity of mortality estimation was related to the likelihood of an individual to die at hospital. While the majority of French die in the hospital (57.4%, see below), bias may be introduced especially if the people dying in a hospital are different from the people dying outside. If dying inside or outside the hospital is a random process then our sample did not suffer from selection bias for mortality estimation (i.e., the absolute numbers will be underestimated, but the relationships will be correct). Otherwise, if the place of death is related to health status, then dedicated analyses may be necessary to exclude such bias. While we cannot control for health status directly, we examined the likelihood of bias via a proxy variable of health status, premature mortality (≤65 years) by gender.

External data sources (National Vital Statistics based on death certificates) indicate that a total of 2,672,906 adults residing in Metropolitan France died between 2008 and 2012. Overall, 1,535,185 (57.4%) deaths were recorded at hospital and thus were covered by our data sources. Premature mortality was more marked in men than women (27.5% of 1,355,892 men versus 13.3% of 1,317,014 women). Death was recorded at hospital in about the same proportions for premature and other mortality (58.8% and 57.1%, respectively), indicating no bias. Opposite trends were found by gender (men: 56.1% versus 62.1%; women: 64.7% versus 52.8%) as explained by increasing proportions of deaths recorded in nursing home with an older age. Consequently, all analyses were conducted separately by sex, and sensitivity analyses were performed on the presence of alcohol-attributable disease categories before death depending on premature mortality.

## Results

### All-cause mortality risk

**Fig. 1:**
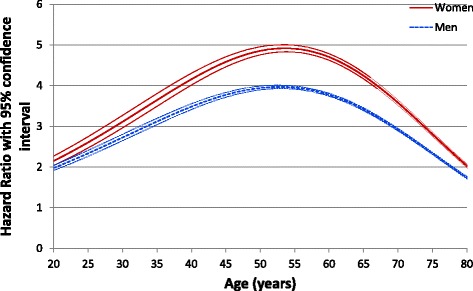
Instantaneous risk of death of people with alcoholic use disorders over lifetime (French National Hospital Discharge database 2008–2012)

Table 1 gives an overview of baseline characteristics of the sample, prevalence of AUD and associated in-hospital death rates. Overall, 704,803 (2.7%) patients identified with AUDs had a threefold higher risk of death (hazard ratio (HR) = 2.98, 95% CI: 2.96–3.00) compared to all other adults discharged from French hospitals in 2008–2012. One-quarter of patients with AUDs were women with a higher risk of death (HR = 3.30, 95% CI: 3.25–3.34 compared to HR = 2.90, 95% CI: 2.88–2.92 for men). The instantaneous risk of death with AUDs was not proportional over lifetime: it increased sharply from age 20 to 53 (2.15 to 4.92 in women; 1.98 to 3.97 in men) and remained significantly above 1 until age 86 (Fig. 1).

**Table 1:** Baseline Characteristics of the Adult Population Residing in Metropolitan France and Discharged from Hospitals in 2008–2012

Characteristics	All Adults		Men		Women	
	n (%)	In-Hospital Death, n (%)	n (%)	In-Hospital Death, n (%)	n (%)	In-Hospital Death, n (%)
	26,356,361 (100)	1,506,334 (5.7)	12,300,516 (46.7)	816,259 (6.6)	14,055,845 (53.3)	690,075 (4.9)
Age at cohort inception (Jan. 1st, 2008) in years						
< 30	4,687,102 (17.8)	11,077 (0.2)	2,255,316 (18.3)	7053 (0.3)	2,431,786 (17.3)	4024 (0.2)
30–39	3,206,919 (12.2)	19,546 (0.6)	1,476,922 (12.0)	10,888 (0.7)	1,729,997 (12.3)	8658 (0.5)
40–49	4,007,783 (15.2)	67,554 (1.7)	1,902,807 (15.5)	40,517 (2.1)	2,104,976 (15.0)	27,037 (1.3)
50–59	4,658,741 (17.7)	167,946 (3.6)	2,336,423 (19.0)	111,321 (4.8)	2,322,318 (16.5)	56,625 (2.4)
60–69	3,782,817 (14.4)	222,356 (5.9)	1,894,887 (15.4)	145,912 (7.7)	1,887,930 (13.4)	76,444 (4.1)
70–79	3,603,656 (13.7)	426,766 (11.8)	1,605,710 (13.1)	247,181 (15.4)	1,997,946 (14.2)	179,585 (9.0)
80–89	2,108,262 (8.0)	491,158 (23.3)	754,991 (6.1)	223,228 (29.6)	1,353,271 (9.6)	267,930 (19.8)
≥ 90	301,081 (1.1)	99,931 (33.2)	73,460 (0.6)	30,159 (41.1)	227,621 (1.6)	69,772 (30.7)
Region of residency in Metropolitan France						
Greater Paris area	4,450,245 (16.9)	217,573 (4.9)	2,014,604 (16.4)	113,952 (5.7)	2,435,641 (17.3)	103,621 (4.3)
North-West	6,051,875 (23.0)	373,476 (6.2)	2,849,253 (23.2)	206,088 (7.2)	3,202,622 (22.8)	167,388 (5.2)
North-East	6,023,280 (22.9)	372,947 (6.2)	2,806,326 (22.8)	200,203 (7.1)	3,216,954 (22.9)	172,744 (5.4)
South-West	3,080,671 (11.7)	175,921 (5.7)	1,452,170 (11.8)	95,699 (6.6)	1,628,501 (11.6)	80,222 (4.9)
South-East	6750,290 (25.6)	366,417 (5.4)	3,178,163 (25.8)	200,317 (6.3)	3,572,127 (25.4)	166,100 (4.7)
Tertiary care center	6,458,321 (24.5)	595,893 (9.2)	3,040,223 (24.7)	337,187 (11.1)	3,418,098 (24.3)	258,706 (7.6)
Alcohol Use Disorders	704,803 (2.7)	112,752 (16.0)	528,480 (4.3)	89,165 (16.9)	176,323 (1.3)	23,587 (13.4)

### Cause-specific risks of hospital admission

Tables 2 and 3 give the hazard ratios for alcohol-attributable disease categories in men and women, respectively. In both gender, AUDs were associated with significantly higher risks for all alcohol-attributable disease categories, including colorectal cancer but not breast cancer.

**Table 2:** Hazard Ratios for Alcohol-Attributable Disease Categories in 12,300,516 Men (French National Hospital Discharge database 2008–2012)

Alcohol-Attributable Disease Category	Total, n (%)	AUDs, n (%)	Mean (SD) age at incidence		Hazard Ratio with AUDs (95% CI)
			AUDs	No AUDs	
Liver diseases					
Cirrhosis	357,809 (2.91)	155,000 (43.3)	59.6 (12.0)	64.9 (16.6)	15.97 (15.86–16.09)
Liver cancer	56,162 (0.46)	26,199 (46.7)	65.3 (9.9)	69.6 (12.4)	16.12 (15.84–16.40)
Pancreatic diseases					
Acute pancreatitis	80,860 (0.66)	32,711 (40.5)	48.8 (13.2)	60.7 (18.0)	12.14 (11.96–12.32)
Chronic pancreatitis	57,465 (0.47)	31,602 (55.0)	53.2 (12.3)	62.3 (16.1)	20.36 (20.01–20.71)
Pancreatic cancer	41,853 (0.34)	3659 (8.7)	62.2 (10.9)	68.6 (12.0)	1.61 (1.56–1.67)
Upper aerodigestive tract cancers					
Head and neck cancer	82,852 (0.67)	19,875 (24.0)	57.8 (8.7)	63.2 (11.9)	4.39 (4.31–4.46)
Larynx cancer	37,952 (0.31)	8399 (22.1)	59.0 (8.9)	64.9 (11.1)	4.03 (3.93–4.14)
Oesophageal cancer	36,083 (0.29)	6084 (16.9)	60.7 (9.4)	66.8 (11.5)	3.19 (3.10–3.28)
Colorectal cancer	190,593 (1.55)	10,427 (5.5)	65.7 (10.3)	69.5 (12.2)	1.14 (1.11–1.16)
Cardiovascular diseases					
Ischemic heart disease	1,290,533 (10.49)	71,137 (5.5)	62.6 (11.5)	68.8 (12.8)	1.11 (1.10–1.12)
Congestive heart failure	980,140 (8.0)	76,602 (7.8)	64.4 (12.1)	73.4 (12.8)	1.95 (1.94–1.97)
Atrial fibrillation	898,455 (7.30)	54,639 (6.1)	67.5 (11.5)	74.4 (11.9)	1.64 (1.63–1.66)
Ischemic stroke	432,393 (3.52)	34,288 (7.9)	63.8 (11.4)	71.3 (13.0)	1.75 (1.73–1.77)
Haemorrhagic and other non-ischemic stroke	108,219 (0.88)	12,164 (11.2)	59.6 (12.7)	67.9 (16.9)	2.23 (2.19–2.28)
Dementia	389,687 (3.17)	47,349 (12.2)	65.6 (13.2)	80.5 (9.1)	4.67 (4.63–4.72)
Infectious diseases					
Pneumococcal pneumonia	28,569 (0.23)	4799 (16.8)	56.4 (12.9)	64.7 (18.6)	3.71 (3.60–3.83)
Tuberculosis	42,945 (0.35)	4524 (10.5)	57.4 (13.3)	61.8 (20.3)	2.22 (2.15–2.29)
Injuries					
Non-intentional injury	357,461 (2.91)	42,425 (11.9)	56.7 (15.6)	56.5 (24.1)	3.11 (3.08–3.15)
Self-harm	144,723 (1.18)	36,548 (25.3)	43.1 (12.0)	39.4 (17.1)	6.55 (6.47–6.64)
Other intentional injury	32,113 (0.26)	5966 (18.6)	43.7 (21.9)	36.7 (18.3)	5.02 (4.88–5.17)

**Table 3:** Hazard Ratios for Alcohol-Attributable Disease Categories in 14,055,845 Women (French National Hospital Discharge database 2008–2012)

Alcohol-Attributable Disease Category	Total, n (%)	AUDs, n (%)	Mean (SD) age at incidence		Hazard Ratio with AUDs (95% CI)
			AUDs	No AUDs	
Liver diseases					
Cirrhosis	250,148 (1.78)	52,348 (20.9)	60.2 (12.5)	65.6 (18.3)	19.28 (10.09–19.47)
Liver cancer	21,171 (0.15)	3690 (17.4)	66.4 (11.2)	71.7 (13.8)	13.24 (12.76–13.73)
Pancreatic diseases					
Acute pancreatitis	59,304 (0.42)	6737 (11.4)	50.4 (14.2)	61.1 (20.9)	8.56 (8.34–8.78)
Chronic pancreatitis	32,289 (0.23)	6490 (20.1)	54.0 (13.3)	64.2 (17.8)	15.36 (14.93–15.80)
Pancreatic cancer	38,627 (0.23)	858 (2.2)	63.6 (12.5)	72.0 (13.2)	1.44 (1.35–1.54)
Upper aerodigestive tract cancers					
Head and neck cancer	24,814 (0.18)	3255 (13.1)	56.9 (9.0)	65.3 (14.9)	7.54 (7.26–7.83)
Larynx cancer	5267 (0.04)	924 (17.5)	57.6 (8.7)	63.9 (13.0)	9.68 (9.00–10.41)
Esophageal cancer	9399 (0.07)	926 (9.9)	61.0 (9.6)	69.8 (13.2)	6.23 (5.81–6.67)
Colorectal cancer	165,510 (1.18)	2584 (1.6)	67.3 (11.1)	71.2 (13.7)	1.09 (1.05–1.14)
Breast cancer	461,426 (3.28)	6108 (1.3)	62.3 (11.9)	62.6 (14.4)	0.65 (0.63–0.67)
Cardiovascular diseases					
Ischemic heart disease	717,549 (5.10)	12,170 (1.7)	66.5 (13.1)	76.1 (12.7)	1.45 (1.43–1.48)
Congestive heart failure	829,405 (5.90)	17,822 (2.2)	68.4 (12.9)	80.0 (11.6)	2.35 (2.32–2.39)
Atrial fibrillation	818,585 (5.82)	13,547 (1.7)	71.9 (10.8)	79.9 (10.4)	1.84 (1.81–1.87)
Ischemic stroke	377,120 (2.68)	8562 (2.3)	65.9 (12.9)	75.8 (14.2)	1.92 (1.88–1.97)
Hemorrhagic and other non-ischemic stroke	100,700 (0.72)	3864 (3.8)	61.3 (13.1)	72.0 (16.8)	2.66 (2.58–2.75)
Dementia	675,778 (4.81)	19,720 (2.9)	69.4 (13.3)	83.3 (8.1)	4.27 (4.21–4.33)
Infectious diseases					
Pneumococcal pneumonia	20,642 (0.15)	1193 (5.8)	55.5 (13.5)	67.5 (20.0)	4.35 (4.10–4.62)
Tuberculosis	36,103 (0.26)	889 (2.5)	59.8 (15.0)	65.7 (20.8)	1.80 (1.68–1.92)
Injuries					
Non-intentional injury	404,365 (2.88)	17,364 (4.3)	61.5 (15.2)	71.8 (20.5)	4.49 (4.42–4.56)
Self-harm	223,769 (1.59)	24,893 (11.1)	45.2 (12.2)	40.6 (18.0)	8.90 (8.78–9.03)
Other intentional injury	17,958 (0.13)	2619 (14.6)	46.5 (13.3)	46.5 (22.7)	12.58 (12.05–13.13)

Elevated risks were highest for digestive disease categories, followed by upper aerodigestive tract cancers, dementia, injuries, and infectious diseases. No protective effects was found in any cardiovascular diseases with hazard ratios varying between 1.11 (95% CI: 1.10–1.12) for ischemic heart disease in men and 2.66 (95% CI: 2.58–2.75) for haemorrhagic and non-ischemic stroke in women. Hazard ratios were not proportional over lifetime (Additional file 1: Figs. S1–S21): they were all bell-shaped around an earlier onset of each disease category with exception of ischemic heart disease in men (flat risk over lifetime), colorectal and breast cancers (both showing later onset in AUDs’ survivors).

### Presence of alcohol-attributable disease categories at death and age at death

Table 4 gives all alcohol-attributable disease categories recorded before death and age at death for 1,506,334 adult patients dying at French hospitals in 2008–2012. AUDs were recorded in 112,752 (7.5%) deceased patients (men: 10.9%; women: 3.4%). One or more alcohol-attributable disease categories were found in 93.2% deceased patients with AUDs, with liver diseases recorded in about two-third patients (64.3% in men; 71.1% in women). Similar proportions were found by gender depending on premature mortality (Additional file 1: Tables S2 and S3). Overall, the average age at death was 64.9 years for patients with AUDs, i.e., 12.2 years younger than other adults dying at hospital without AUDs (men: 10.4 years, 95% CI: 10.3–10.5; women: 13.7 years, 95% CI: 13.6–13.9).

**Table 4:** Alcohol-Attributable Disease Categories at Death, by gender and AUDs status (French National Hospital Discharge database 2008–2012)

	Men (*n* = 816,259)				Women (*n* = 690,194)			
	AUDs, n (%)	No AUDs, n (%)	Mean (SD) age at death		AUDs, n (%)	No AUDs, n (%)	Mean (SD) age at death	
			AUDs	No AUDs			AUDs	No AUDs
	89,165 (10.9)	727,094 (89.1)	64.8 (11.7)	75.2 (13.3)	23,587 (3.4)	666,607 (96.6)	65.5 (12.7)	79.2 (13.4)
Alcohol-attributable disease categories								
One or more disease categories	83,039 (93.1)	552,314 (76.0)	65.0 (11.6)	76.7 (12.4)	22,064 (93.5)	503,466 (75.5)	65.8 (12.6)	81.0 (12.1)
Liver diseases	57,298 (64.3)	92,547 (12.7)	64.5 (11.2)	71.1 (13.1)	16,770 (71.1)	82,348 (12.4)	64.7 (12.0)	72.5 (14.1)
Pancreatic diseases	8372 (9.4)	30,672 (4.2)	61.8 (11.9)	71.4 (12.1)	1812 (7.7)	28,149 (4.2)	62.2 (13.3)	75.5 (12.3)
Upper aerodigestive tract cancer	11,552 (13.0)	42,250 (5.8)	61.4 (9.3)	67.9 (11.7)	1685 (7.1)	10,455 (1.6)	60.5 (9.5)	71.2 (13.5)
Colorectal cancer	3952 (4.4)	51,606 (7.1)	68.8 (10.0)	74.5 (11.4)	872 (3.7)	42,223 (6.3)	70.4 (10.5)	76.7 (12.5)
Cardiovascular diseases	39,057 (43.8)	406,023 (55.8)	68.6 (11.2)	78.8 (11.0)	9291 (39.4)	362,751 (54.4)	70.5 (12.2)	83.3 (10.2)
Dementia	13,557 (15.2)	102,203 (14.1)	69.7 (12.1)	83.3 (7.7)	4406 (18.7)	129,161 (19.4)	71.5 (12.7)	86.1 (7.2)
Infectious diseases	2384 (2.7)	14,298 (2.0)	63.3 (12.2)	75.5 (12.8)	509 (2.2)	9751 (1.5)	63.5 (13.6)	79.6 (12.5)
Non-intentional injury	6750 (7.6)	41,903 (5.8)	67.7 (12.4)	79.2 (13.4)	2280 (9.7)	49,764 (7.5)	69.8 (12.5)	83.8 (10.4)
Intentional injury	2019 (2.3)	5822 (0.8)	60.0 (13.3)	64.4 (20.2)	881 (3.7)	4824 (0.7)	56.2 (12.6)	68.0 (19.0)
None of the above	6126 (6.9)	174,780 (24.0)	61.0 (11.6)	70.3 (14.9)	1523 (6.5)	163,141 (24.5)	61.0 (12.2)	73.6 (15.6)
Breast cancer (Women)					1412 (6.0)	62,449 (9.4)	67.2 (11.4)	70.8 (14.4)

## Discussion

AUDs were associated with marked premature mortality and higher risks of hospitalization for alcohol-attributable disease categories. The hazard ratio for all-cause mortality was threefold (HR = 2.98; 95% CI: 2.96–3.00), with patients with AUDs dying on average 12.2 years younger (men: 10.4, 95% CI: 10.3–10.5; women: 13.7, 95% CI: 13.6–13.9). It should be noted that the relative risks and life expectancy measures were in comparison to other people visiting the hospital, whereas many prior studies used standardized mortality ratios, which would include the whole population including those who did not go to the hospital during the period examined [28, 29, 32, 33]. Despite this difference, the relative risks found are comparable to the relative risks found in the meta-analyses of studies based on standardized mortality ratios [29, 32].

Our study found higher relative all-cause mortality risk for women with AUDs than for men, even though their drinking level has been shown to be lower in terms of average consumption of pure alcohol per day [34, 35]. The reason for this can be found in the different alcohol metabolism, leading to higher blood alcohol concentration in women [36]. Elevated risks of hospital admission were highest for liver diseases that eventually accounted for about two-third of deaths recorded among patients with AUDs. AUDs were associated with significantly higher risks of hospital admission for all other alcohol-attributable disease categories except breast cancer. This confirms scarce results from earlier studies [28, 29], including results on increased risks for ischemic heart disease and other cardiovascular risk factors in a nationwide study [14]. Among patients with AUDs, any hospital record of abstinence was associated with a substantially reduced all-cause mortality risk by 34.5% (95% CI: 33.3–35.7).

The French healthcare system provides universal access to hospital care. This national retrospective cohort study eventually involved 51.9% of the total adult population residing in Metropolitan France by January 2008. It is by far the largest and most complete study relating AUDs to disease and injury outcomes. The French National Hospital Discharge database has been shown to be complete and of good quality [30]. In particular, records of in-hospital deaths are close between the National Hospital Discharge database and National Vital Statistics based on death certificates, and account for about 57% of all deaths in 2008–2012 in France [37, 38]. Deaths outside the hospital may be seen as a limitation of the study, as it cannot be excluded, that systematic bias had been introduced this way. However, the majority of deaths were in the hospital in all age groups, with no indication of a strong bias by age (see Additional file 1: Tables S2 and S3).

A weakness is the operationalization of the independent variable, AUDs. As this operationalization is based on discharge records, it can only measure AUDs which were recognised and assessed at hospital. Given that AUDs are highly stigmatized [39] with low treatments rates in Europe (around 10% in recent studies [4, 40]), it is likely that only the more severe AUDs were identified, which would mean that the overall impact of AUDs and heavy drinking was underestimated in our analyses. This conclusion is also likely comparing the proportion of adults with AUDs in the hospital discharge database (2.7%) with the prevalence estimated in epidemiological studies (estimate of 6% for France; 3.4% for alcohol dependence alone [41]).

As indicated above, the current study is by far the largest study on the risks for diseases subsequent to AUDs. In general, results corroborated the hypotheses based on epidemiological studies for heavy drinking, with exception of breast cancer. This latter result is puzzling as alcohol consumption has been shown to impact on breast cancer consistently over adult life-course in a dose-response relationship [42, 43]. However, the relative risks found in meta-analyses and large studies for breast cancer were among the lowest of all alcohol-attributable cancers (most risks for heavy drinking around 1.5 [43, 44]). As evidenced by our study, the high level of competing risks in patients with AUDs, i.e. other alcohol-attributable conditions and premature death, and the long lag time for developing breast cancer seem to be an explanation.

Results showed, that co-morbidity of alcohol-attributable diseases was high at death. However, potential interactions between AUD and other co-morbid disorders on risk have not been modelled in the current study.

Since the burden associated with AUDs has been re-confirmed in this study, efforts should be undertaken to reduce this burden. Most importantly, treatment (alcohol rehabilitation) reduced this burden regardless of long-term abstinence (see also [45] for a similar finding in a meta-analysis of mortality after reduction of drinking in AUDs). A clear implication of our study is thus to increase the treatment rate of AUDs which is the lowest for all mental disorders [40]. Currently, most recommendations about screening for alcohol are for primary health care [46]. Given the above results, these recommendations should be supplemented by better screening, interventions, and referral from acute hospitals to specialised care, or by incorporated addictions specialist services into hospitals. A Cochrane review has shown that even brief interventions in the hospital setting could reduce the one year mortality rate by 40% [47]. Being a patient in a hospital seems to be a critical moment, where awareness of the problem and motivation to reduce or abstain from alcohol consumption can be initiated.

Research should be conducted to better integrate screening, brief interventions, and referral into the clinical routine of acute care hospitals. Screening may be linked to routine work of nurses [48], but it will need protected time within the routine, financial compensation, and adequate training [49]. Implementation trials are needed to identify the best strategy to achieve these goals. Finally, given the large overall impact, proven alcohol policy measures to reduce the harmful effects of drinking should be reinforced [50, 51].

## Conclusions

AUD were highly associated with hospitalizations and premature death in most disease and injury categories which had been identified as partially caused by alcohol use, creating a  high burden.  Interventions should be implemented to reduce this burden.

## Additional file

Additional file 1: Additional Tables and Figures for Alcohol use disorders and associated chronic disease – a national retrospective cohort study from France. (DOCX 1775 kb)

## Abbreviations

AUD: Alcohol use disorders
CI: Confidence interval
HR: Hazard ratio
ICD: WHO International Classification of Diseases
n: number of people
SD: Standard deviation
