# Supplementary material for: Alcohol use disorders and associated chronic disease – a national retrospective cohort study from France
Source: BMC Public Health. 2017 Jul 21;18:43. doi: 10.1186/s12889-017-4587-y (PMC5521064; doi:10.1186/s12889-017-4587-y)
Supplement: Additional file 1: — Additional Tables and Figures for Alcohol use disorders and associated chronic disease – a national retrospective cohort study from France. (DOCX 1775 kb) [file 12889_2017_4587_MOESM1_ESM.docx]

**Additional file 1**

**Alcohol use disorders and associated chronic disease –
a national retrospective cohort study from France.**

Michaël Schwarzinger, Sophie Pascale Thiébaut, Sylvain Baillot, Vincent Mallet, Jürgen Rehm

Content

[Table S1: ICD-10 code dictionary 3](#_Toc471102520)

[Table S2. Presence of Alcohol-Attributable Disease Categories in 816,259 Deceased Men, by Premature Death and AUDs status (French National Hospital Discharge database 2008-2012) 4](#_Toc471102521)

[Table S3. Presence of Alcohol-attributable disease categories in 690,194 deceased women at hospital, by premature death and AUD status (French National Hospital Discharge database 2008-2012) 5](#_Toc471102522)

[Figure S1. Instantaneous Risk of Cirrhosis with Alcoholic Use Disorders over the lifetime (French National Hospital Discharge database 2008-2012) 6](#_Toc471102523)

[Figure S2. Instantaneous Risk of Primary Liver Cancer with Alcoholic Use Disorders over the lifetime (French National Hospital Discharge database 2008-2012) 7](#_Toc471102524)

[Figure S3. Instantaneous Risk of Acute Pancreatitis with Alcoholic Use Disorders over the lifetime (French National Hospital Discharge database 2008-2012) 8](#_Toc471102525)

[Figure S4. Instantaneous Risk of Chronic Pancreatitis with Alcoholic Use Disorders over the lifetime (French National Hospital Discharge database 2008-2012) 9](#_Toc471102526)

[Figure S5. Instantaneous Risk of Pancreatic Cancer with Alcoholic Use Disorders over the lifetime (French National Hospital Discharge database 2008-2012) 10](#_Toc471102527)

[Figure S6. Instantaneous Risk of Head and Neck Cancer with Alcoholic Use Disorders over the lifetime (French National Hospital Discharge database 2008-2012) 11](#_Toc471102528)

[Figure S7. Instantaneous Risk of Laryngeal Cancer with Alcoholic Use Disorders over the lifetime (French National Hospital Discharge database 2008-2012) 12](#_Toc471102529)

[Figure S8. Instantaneous Risk of Oesophageal Cancer with Alcoholic Use Disorders over the lifetime (French National Hospital Discharge database 2008-2012) 13](#_Toc471102530)

[Figure S9. Instantaneous Risk of Colorectal Cancer with Alcoholic Use Disorders over the lifetime (French National Hospital Discharge database 2008-2012) 14](#_Toc471102531)

[Figure S10. Instantaneous Risk of Breast Cancer with Alcoholic Use Disorders over the lifetime (French National Hospital Discharge database 2008-2012) 15](#_Toc471102532)

[Figure S11. Instantaneous Risk of Ischemic Heart Disease with Alcoholic Use Disorders over the lifetime (French National Hospital Discharge database 2008-2012) 16](#_Toc471102533)

[Figure S12. Instantaneous Risk of Congestive Heart Failure with Alcoholic Use Disorders over the lifetime (French National Hospital Discharge database 2008-2012) 17](#_Toc471102534)

[Figure S13. Instantaneous Risk of Atrial Fibrillation with Alcoholic Use Disorders over the lifetime (French National Hospital Discharge database 2008-2012) 18](#_Toc471102535)

[Figure S14. Instantaneous Risk of Ischemic Stroke with Alcoholic Use Disorders over the lifetime (French National Hospital Discharge database 2008-2012) 19](#_Toc471102536)

[Figure S15. Instantaneous Risk of Haemorrhagic Stroke with Alcoholic Use Disorders over the lifetime (French National Hospital Discharge database 2008-2012) 20](#_Toc471102537)

[Figure S16. Instantaneous Risk of Dementia with Alcoholic Use Disorders over the lifetime (French National Hospital Discharge database 2008-2012) 21](#_Toc471102538)

[Figure S17. Instantaneous Risk of Pneumococcal Pneumonia with Alcoholic Use Disorders over the lifetime (French National Hospital Discharge database 2008-2012) 22](#_Toc471102539)

[Figure S18. Instantaneous Risk of Tuberculosis with Alcoholic Use Disorders over the lifetime (French National Hospital Discharge database 2008-2012) 22](#_Toc471102540)

[Figure S19. Instantaneous Risk of Non-Intentional Injury with Alcoholic Use Disorders over the lifetime (French National Hospital Discharge database 2008-2012) 24](#_Toc471102541)

[Figure S20. Instantaneous Risk of Self-Harm with Alcoholic Use Disorders over the lifetime (French National Hospital Discharge database 2008-2012) 25](#_Toc471102542)

[Figure S21. Instantaneous Risk of Other Violent Injury with Alcoholic Use Disorders over the lifetime (French National Hospital Discharge database 2008-2012) 26](#_Toc471102543)

# Table S1: ICD-10 code dictionary

|  | | **ICD-10 (2008 French version)** | **Alcohol label in ICD-10** | **Reference** |
| --- | --- | --- | --- | --- |
| **Alcohol use disorders (AUDs)** | | | | |
| Harmful use of alcohol | | F10.1 | Yes | [[1](#_ENREF_1)] |
| Alcohol dependence | | F10.24 ; F10.25 ; F10.26 ; F10.3 ; F10.4 ; Z50.2 ; Z71.4 ; Z72.1 | Yes |  |
| Alcohol-related diseases | | E24.4; E51.1; K70x; G31.2; G62.1; G72.1; I42.6; K29.2; K85.2; K86.0 | Yes |  |
| Mental and behavioural disorder due to alcohol use | | F10.5 ; F10.6 ; F10.7 ; F10.8 ; F10.9 | Yes |  |
| Alcohol abstention | | F10.20 ; F10.21 ; F10.22 ; F10.23 | Yes |  |
| **Liver diseases** | | | | |
|  | Cirrhosis | I85.9; I86.4; I98.2; I98.29; K70.3; K71.7; K74.3; K74.4; K74.5; K74.6; K76.6 | Yes | [[2](#_ENREF_2)] |
|  | Decompensated cirrhosis | G92x ; G93.4; I28.0; I85.0; I98.3; I98.20; J94.8; K70.4; K71.1; K72x; K76.7; R17; R18 | No |  |
|  | Liver cancer | C22x | No | [[3](#_ENREF_3)] |
| **Pancreatic diseases** | | | | |
|  | Acute pancreatitis | K85x | Yes | [[4](#_ENREF_4)] |
|  | Chronic pancreatitis | K86x | Yes | [[4](#_ENREF_4)] |
|  | Pancreas cancer | C25x | No | [[3](#_ENREF_3)] |
| **Upper aerodigestive tract cancers** | | | | |
|  | Head and neck cancer (oral cavity, oropharynx, hypopharynx) | C0xx ; C10x ; C12x ; C13x ; C14x | No | [[3](#_ENREF_3)] |
|  | Larynx cancer | C32x | No | [[3](#_ENREF_3)] |
|  | Esophageal cancer | C15x | No | [[3](#_ENREF_3)] |
| **Other cancer** | | | | |
|  | Colorectal cancer (colon, rectum, anus) | C18x; C19x; C20x; C21x | No | [[3](#_ENREF_3)] |
|  | Breast cancer | C50x | No | [[3](#_ENREF_3)] |
| **Cardiovascular diseases** | | | | |
|  | Ischemic heart disease | I20x ; I21x ; I22x ; I23x ; I24x ; I25x | No | [[4](#_ENREF_4)] |
|  | Congestive heart failure | I09.9 ; I11.0 ; I13.0 ; I13.2 ; I25.5 ; I42.0 ; I42.5 ; I42.6 ; I42.7 ; I42.8 ; I42.9 ; I43x ; I50x ; P29.0 | Yes | [[2](#_ENREF_2)] |
|  | Atrial fibrillation | I48x | No | [[4](#_ENREF_4)] |
|  | Ischemic stroke | I63x ; I65x ; I66x ; I67.0 ; I67.1 ; I67.2 ; I67.3 ; I67.5 ; I67.6 ; I67.7 ; I67.8 ; I67.9 ; I69.3 | No | [[4](#_ENREF_4)] |
|  | Hemorrhagic and other non-ischemic stroke | I60x ; I61x ; I62x ; I67.4 ; I69.0 ; I69.1 ; I69.2 | No | [[4](#_ENREF_4)] |
| **Dementia** | | F00x ; F01x ; F02x ; F03x ; F05.1 ; G30x ; G31x | Yes | [[4](#_ENREF_4)] |
| **Infectious diseases** | | | | |
|  | Pneumococcal pneumonia | J13x | No | [[4](#_ENREF_4)] |
|  | Tuberculosis | A1x ; B90x ; P37.0 | No | [[4](#_ENREF_4)] |
| **Injuries** | |  |  |  |
|  | Non-intentional injury | Vxx ; Wxx; X0x; X1x; X2x; X3x; X4x; X5x; Y85x; Y86x | No | [[4](#_ENREF_4)] |
|  | Self-harm | X6xx ; X7xx ; X80x ; X81x ; X82x ; X83x ; X84x ; Y87.0 | No | [[4](#_ENREF_4)] |
|  | Other intentional injury | X85x ; X86x ; X87x ; X88x ; X89x ; X9xx ; Y0xx ; Y1xx ; Y2xx ; Y3xx ; Y87.1 ; Y87.2 ; Y89x | No | [[4](#_ENREF_4)] |

# Table S2. Presence of Alcohol-Attributable Disease Categories in 816,259 Deceased Men, by Premature Death and AUDs status (French National Hospital Discharge database 2008-2012)

|  | | **In-Hospital Death at Age ≤65**  **(n=202,708; 24.8%)** | | **In-Hospital Death at Age >65**  **(n=613,551; 75.2%)** | |
| --- | --- | --- | --- | --- | --- |
|  |  | **AUDs, n (%)** | **No AUDs, n (%)** | **AUDs, n (%)** | **No AUDs, n (%)** |
|  |  | 47,013 (23.2) | 155,695 (76.8) | 42,152 (6.9) | 571,399 (93.1) |
| **Alcohol-attributable disease categories** | |  |  |  |  |
|  | One or more disease categories | 42,953 (91.4) | 95,964 (61.6) | 40,086 (95.1) | 456,350 (79.9) |
|  | Liver diseases | 30,420 (64.7) | 27,763 (17.8) | 26,878 (63.8) | 64,784 (11.3) |
|  | Pancreatic diseases | 5,265 (11.2) | 9,075 (5.8) | 3,107 (7.4) | 21,597 (3.8) |
|  | Upper aerodigestive tract cancer | 7,930 (16.9) | 18,372 (11.8) | 3,622 (8.6) | 23,878 (4.2) |
|  | Colorectal cancer | 1,462 (3.1) | 10,872 (7.0) | 2,490 (5.9) | 40,734 (7.1) |
|  | Cardiovascular diseases | 15,160 (32.3) | 47,743 (30.7) | 23,897 (56.7) | 358,280 (62.7) |
|  | Dementia | 4,925 (10.5) | 2,610 (1.7) | 8,632 (20.5) | 99,593 (17.4) |
|  | Infectious diseases | 1,364 (2.9) | 2,785 (1.8) | 1,020 (2.4) | 11,513 (2.0) |
|  | Non-intentional injury | 2,886 (6.1) | 5,075 (3.3) | 3,864 (9.2) | 36,828 (6.5) |
|  | Intentional injury | 1,562 (3.3) | 2,694 (1.7) | 457 (1.1) | 3,128 (0.6) |

# Table S3. Presence of Alcohol-attributable disease categories in 690,194 deceased women at hospital, by premature death and AUD status (French National Hospital Discharge database 2008-2012)

|  | | **In-Hospital Death at Age ≤65**  **(n=112,359; 16.3%)** | | **In-Hospital Death at Age >65**  **(n=577,835; 83.7%)** | |
| --- | --- | --- | --- | --- | --- |
|  |  | **AUDs, n (%)** | **No AUDs, n (%)** | **AUDs, n (%)** | **No AUDs, n (%)** |
|  |  | 11,846 (10.5) | 100,513 (89.5) | 11,741 (2.0) | 566,094 (98.0) |
| **Alcohol-attributable disease categories** | |  |  |  |  |
|  | One or more disease categories | 10,836 (91.5) | 54,438 (54.2) | 11,228 (95.6) | 449,028 (79.3) |
|  | Liver diseases | 8,708 (73.5) | 23,210 (23.1) | 8,062 (68.7) | 59,138 (10.5) |
|  | Pancreatic diseases | 1,085 (9.2) | 5,513 (5.5) | 727 (6.2) | 22,636 (4.0) |
|  | Upper aerodigestive tract cancer | 1,202 (10.2) | 3,603 (3.6) | 483 (4.1) | 6,852 (1.2) |
|  | Colorectal cancer | 257 (2.2) | 7,720 (7.7) | 615 (5.2) | 34,503 (6.1) |
|  | Cardiovascular diseases | 3,075 (26.0) | 21,757 (21.7) | 6,216 (52.9) | 340,994 (60.2) |
|  | Dementia | 1,381 (11.7) | 1,685 (1.7) | 3,025 (25.8) | 127,476 (22.5) |
|  | Infectious diseases | 291 (2.5) | 1,207 (1.2) | 218 (1.9) | 8,544 (1.5) |
|  | Non-intentional injury | 834 (7.0) | 2,779 (2.8) | 1,446 (12.3) | 46,985 (8.3) |
|  | Intentional injury | 695 (5.9) | 1,965 (2.0) | 186 (1.6) | 2,859 (0.5) |
| Breast cancer | | 632 (5.3) | 21,606 (21.5) | 780 (6.6) | 40,843 (7.2) |

# Figure S1. Instantaneous Risk of Cirrhosis with Alcoholic Use Disorders over the lifetime (French National Hospital Discharge database 2008-2012)


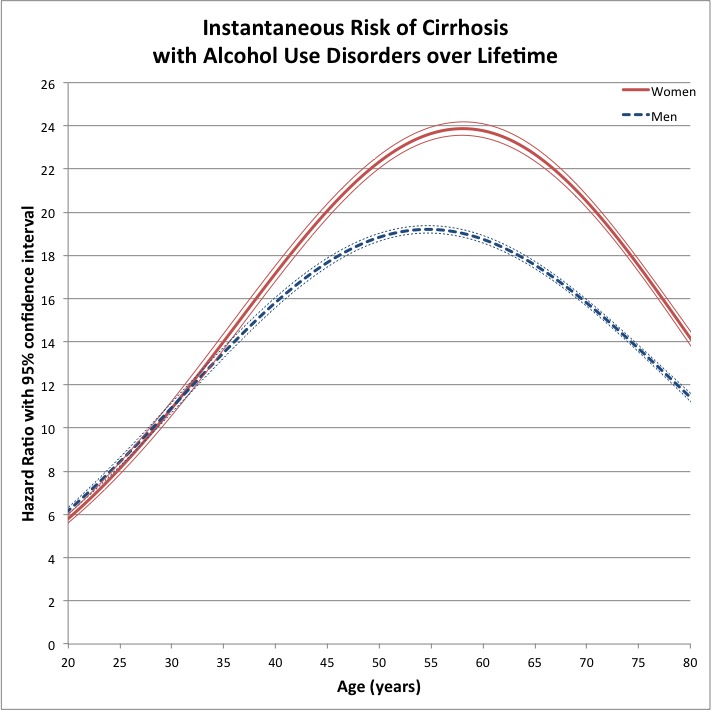


# Figure S2. Instantaneous Risk of Primary Liver Cancer with Alcoholic Use Disorders over the lifetime (French National Hospital Discharge database 2008-2012)


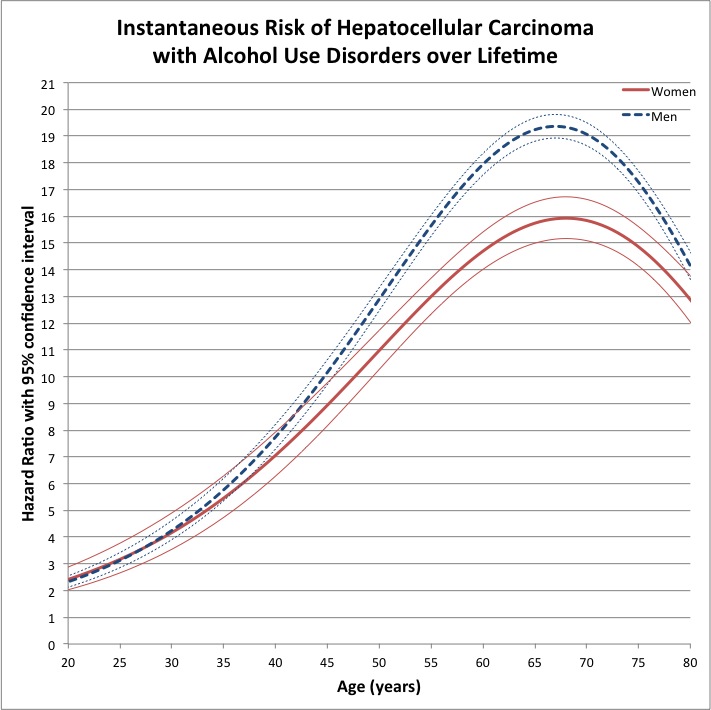


# Figure S3. Instantaneous Risk of Acute Pancreatitis with Alcoholic Use Disorders over the lifetime (French National Hospital Discharge database 2008-2012)


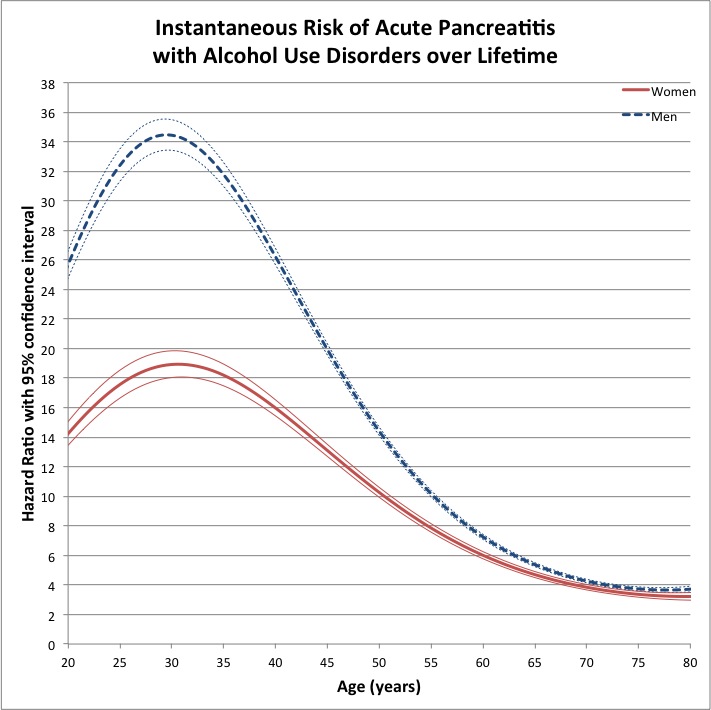


# Figure S4. Instantaneous Risk of Chronic Pancreatitis with Alcoholic Use Disorders over the lifetime (French National Hospital Discharge database 2008-2012)


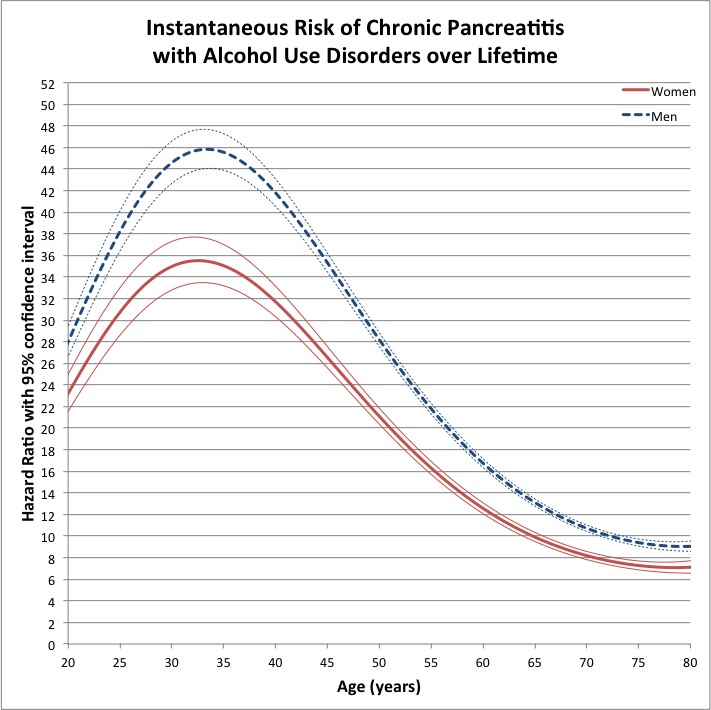


# Figure S5. Instantaneous Risk of Pancreatic Cancer with Alcoholic Use Disorders over the lifetime (French National Hospital Discharge database 2008-2012)


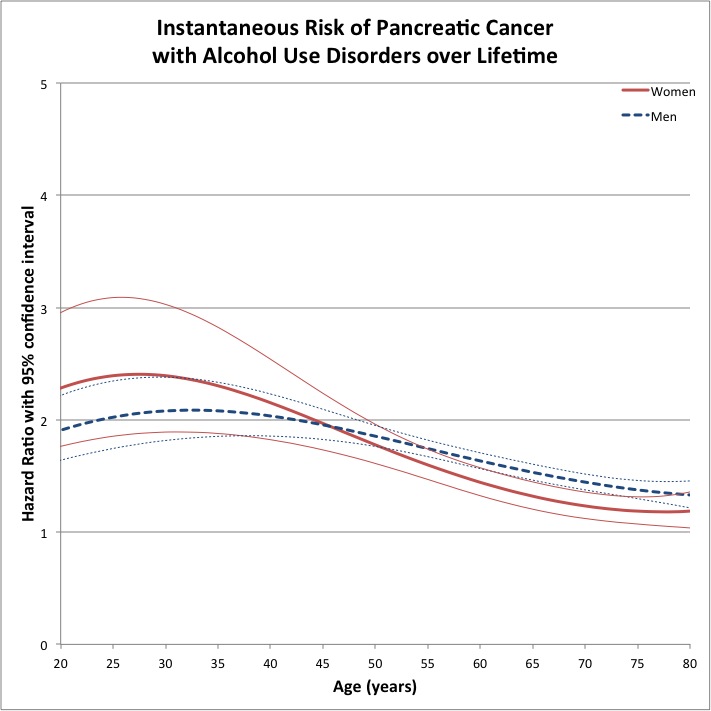


# Figure S6. Instantaneous Risk of Head and Neck Cancer with Alcoholic Use Disorders over the lifetime (French National Hospital Discharge database 2008-2012)


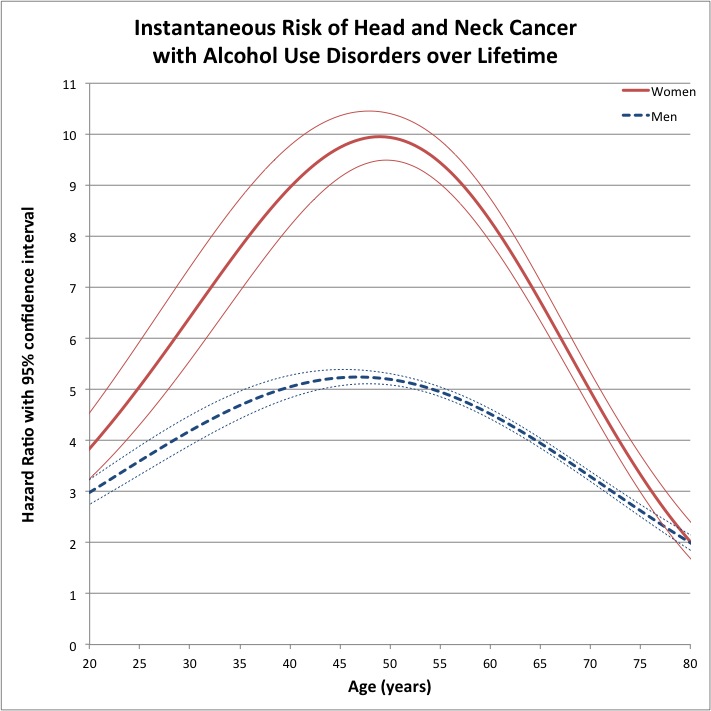


# Figure S7. Instantaneous Risk of Laryngeal Cancer with Alcoholic Use Disorders over the lifetime (French National Hospital Discharge database 2008-2012)


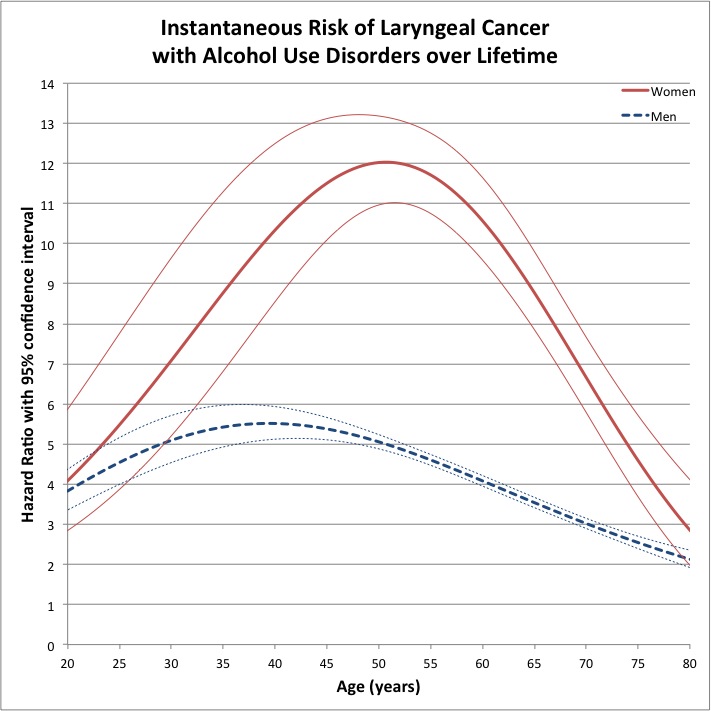


# Figure S8. Instantaneous Risk of Oesophageal Cancer with Alcoholic Use Disorders over the lifetime (French National Hospital Discharge database 2008-2012)


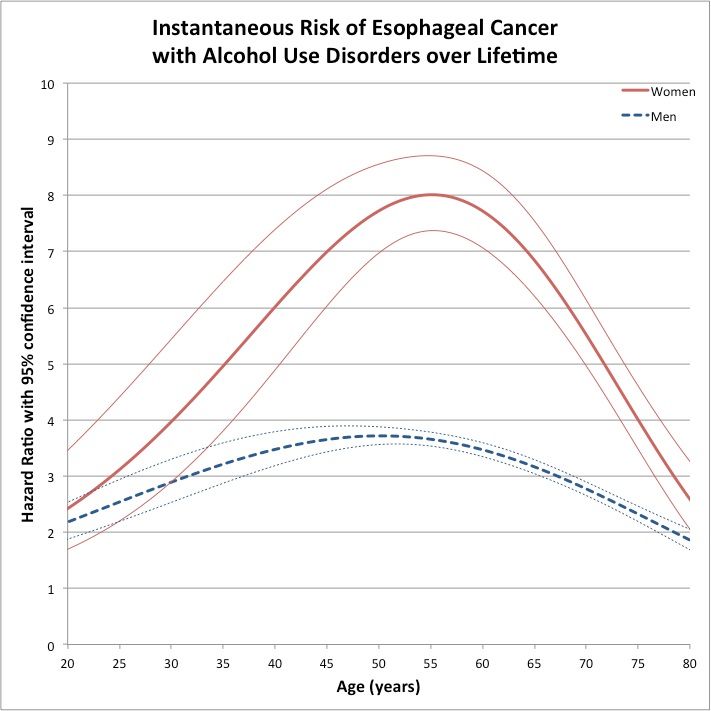


# Figure S9. Instantaneous Risk of Colorectal Cancer with Alcoholic Use Disorders over the lifetime (French National Hospital Discharge database 2008-2012)


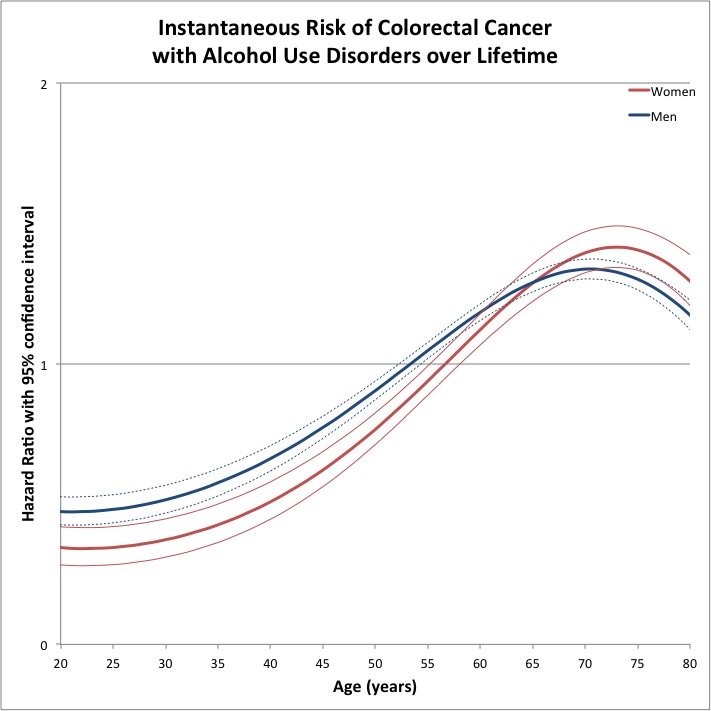


# Figure S10. Instantaneous Risk of Breast Cancer with Alcoholic Use Disorders over the lifetime (French National Hospital Discharge database 2008-2012)


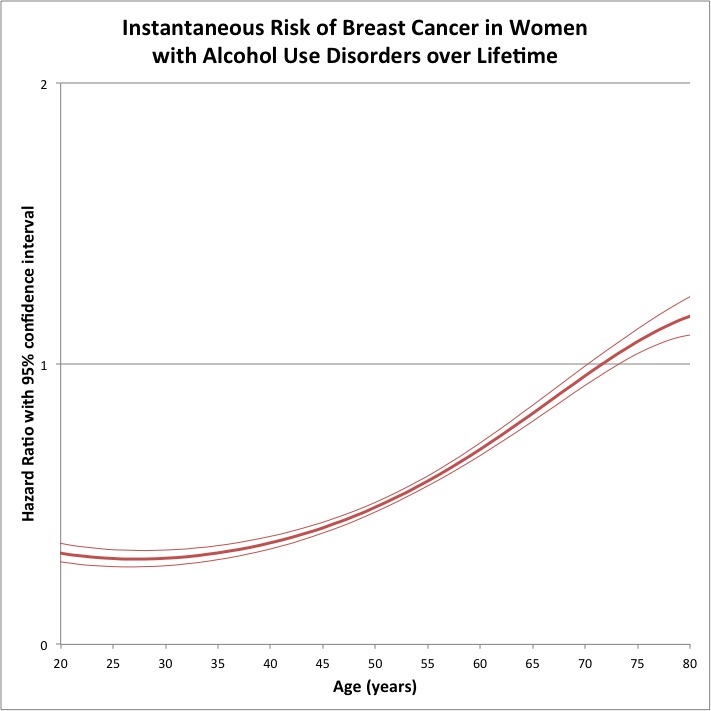


# Figure S11. Instantaneous Risk of Ischemic Heart Disease with Alcoholic Use Disorders over the lifetime (French National Hospital Discharge database 2008-2012)


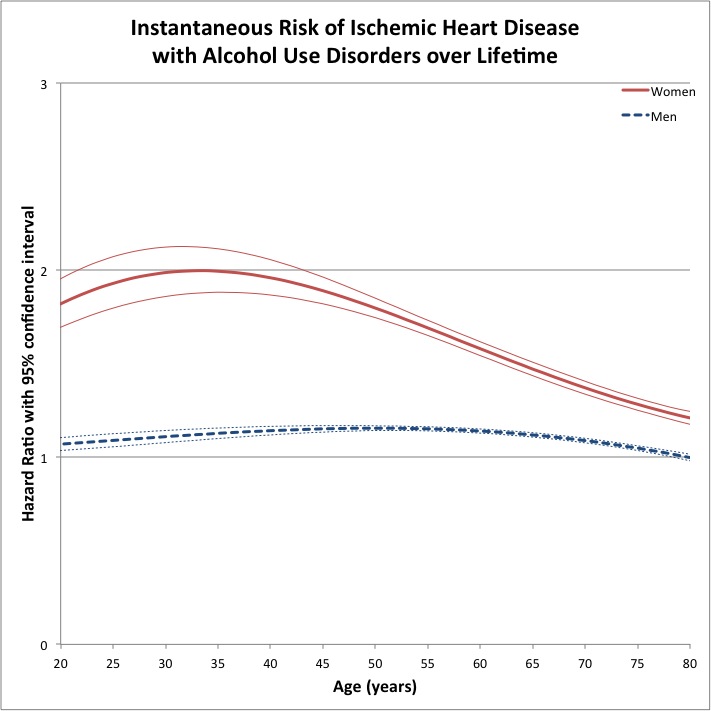


# Figure S12. Instantaneous Risk of Congestive Heart Failure with Alcoholic Use Disorders over the lifetime (French National Hospital Discharge database 2008-2012)


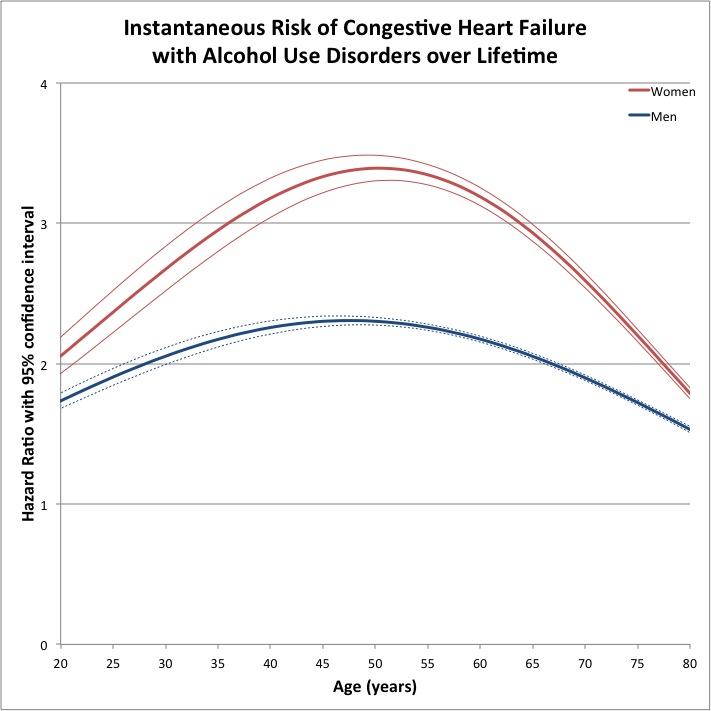


# Figure S13. Instantaneous Risk of Atrial Fibrillation with Alcoholic Use Disorders over the lifetime (French National Hospital Discharge database 2008-2012)


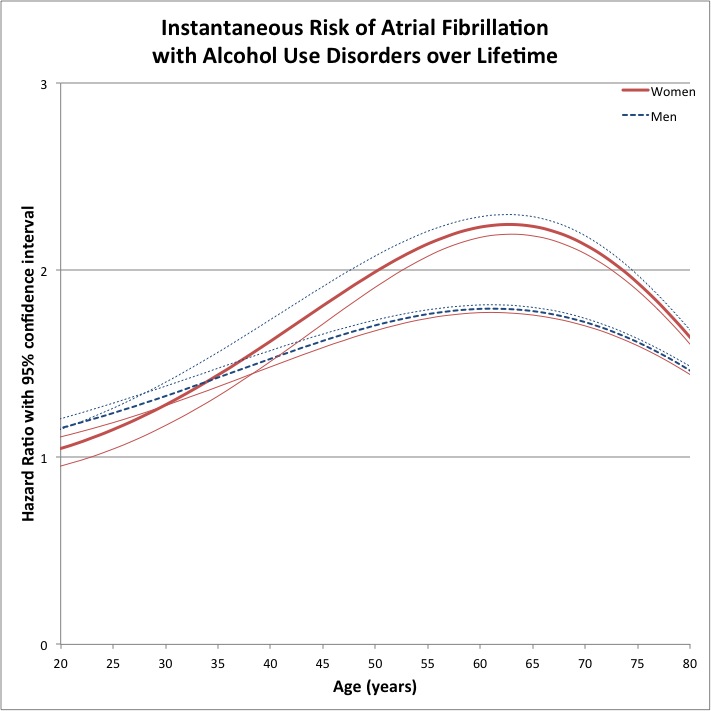


# Figure S14. Instantaneous Risk of Ischemic Stroke with Alcoholic Use Disorders over the lifetime (French National Hospital Discharge database 2008-2012)


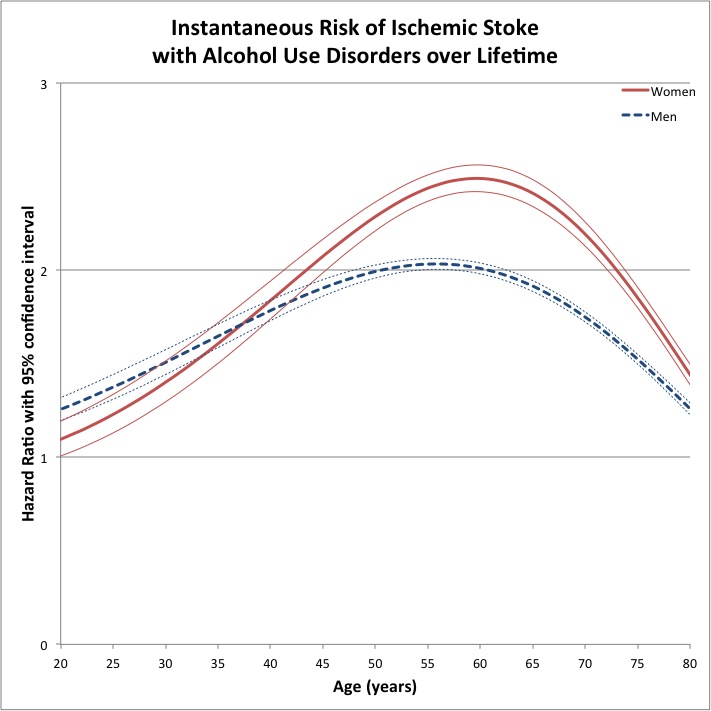


# Figure S15. Instantaneous Risk of Haemorrhagic Stroke with Alcoholic Use Disorders over the lifetime (French National Hospital Discharge database 2008-2012)


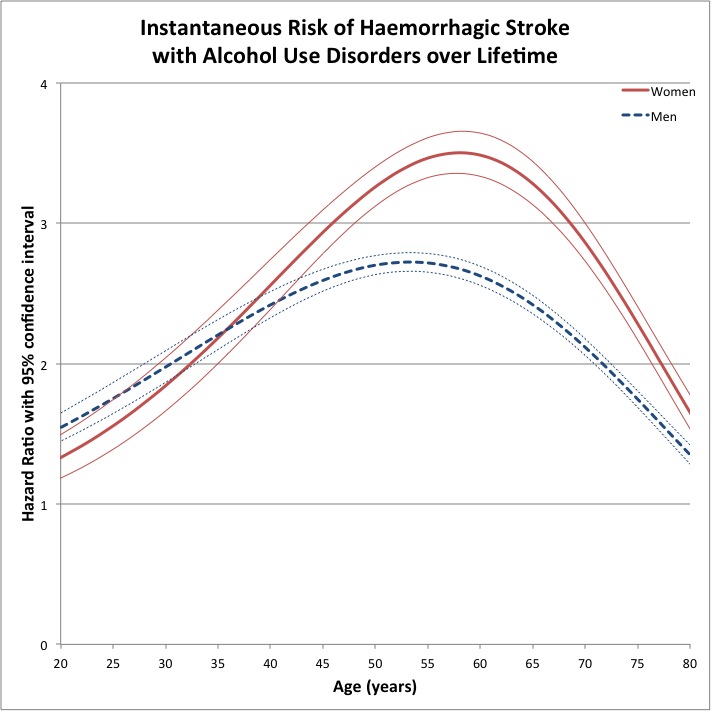


# Figure S16. Instantaneous Risk of Dementia with Alcoholic Use Disorders over the lifetime (French National Hospital Discharge database 2008-2012)


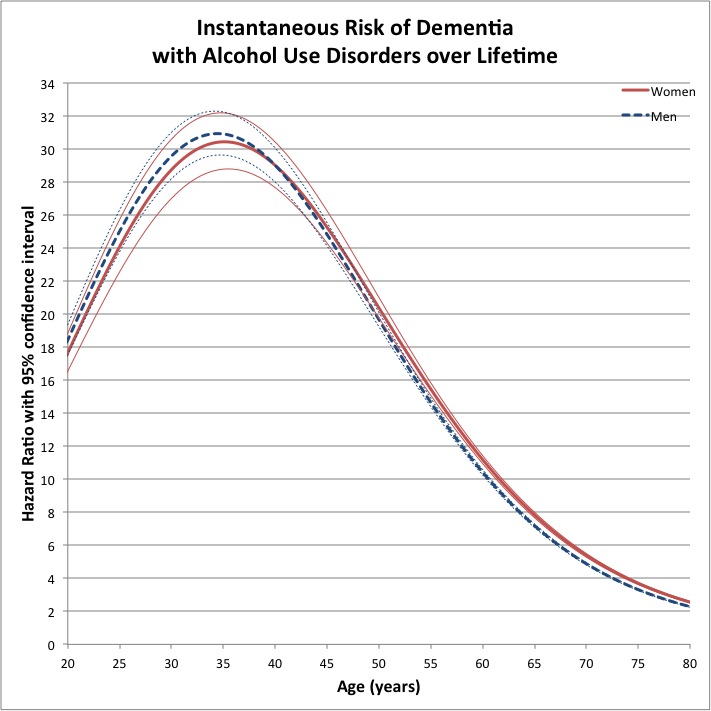


# Figure S17. Instantaneous Risk of Pneumococcal Pneumonia with Alcoholic Use Disorders over the lifetime (French National Hospital Discharge database 2008-2012)


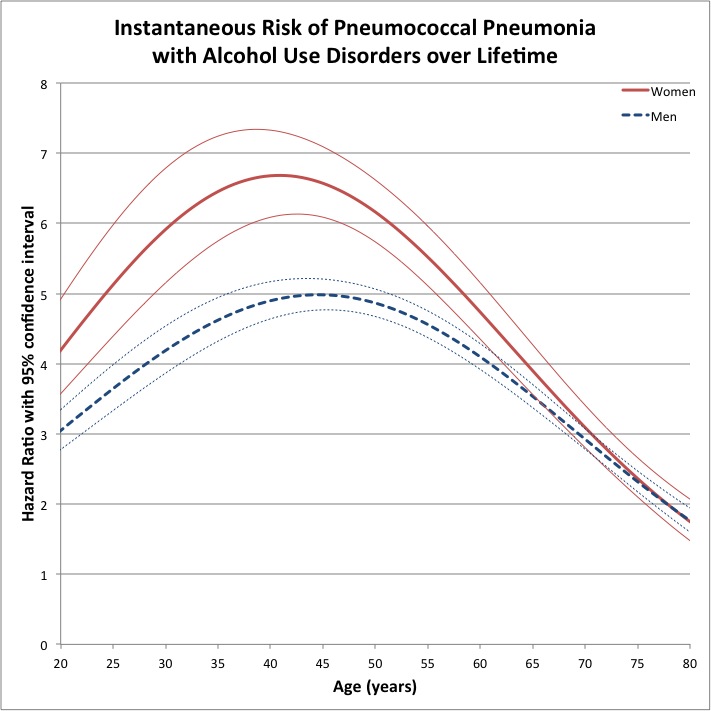


#

# Figure S18. Instantaneous Risk of Tuberculosis with Alcoholic Use Disorders over the lifetime (French National Hospital Discharge database 2008-2012)


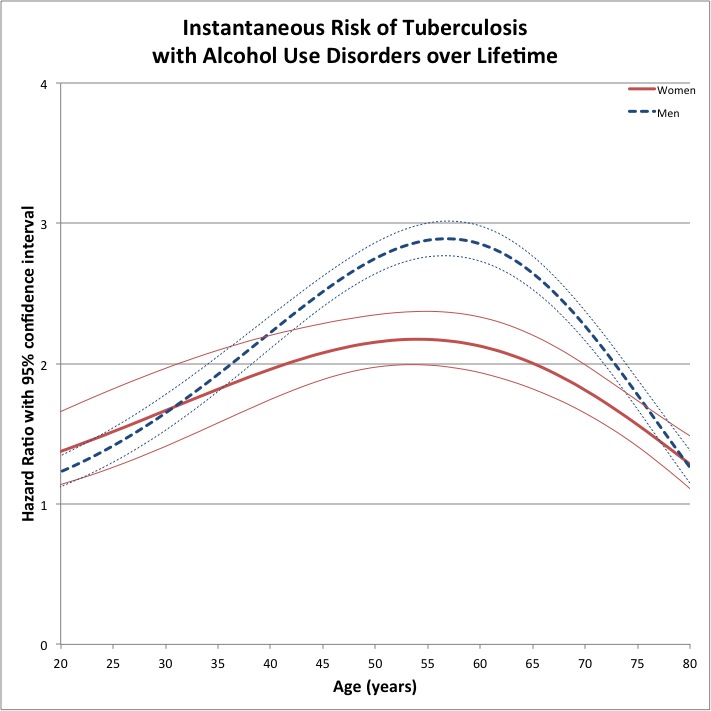


# Figure S19. Instantaneous Risk of Non-Intentional Injury with Alcoholic Use Disorders over the lifetime (French National Hospital Discharge database 2008-2012)


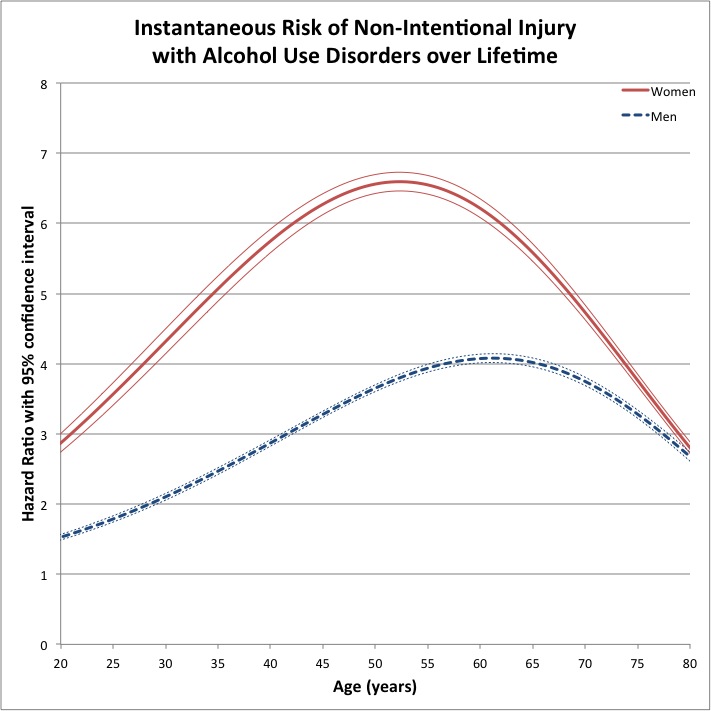


# Figure S20. Instantaneous Risk of Self-Harm with Alcoholic Use Disorders over the lifetime (French National Hospital Discharge database 2008-2012)


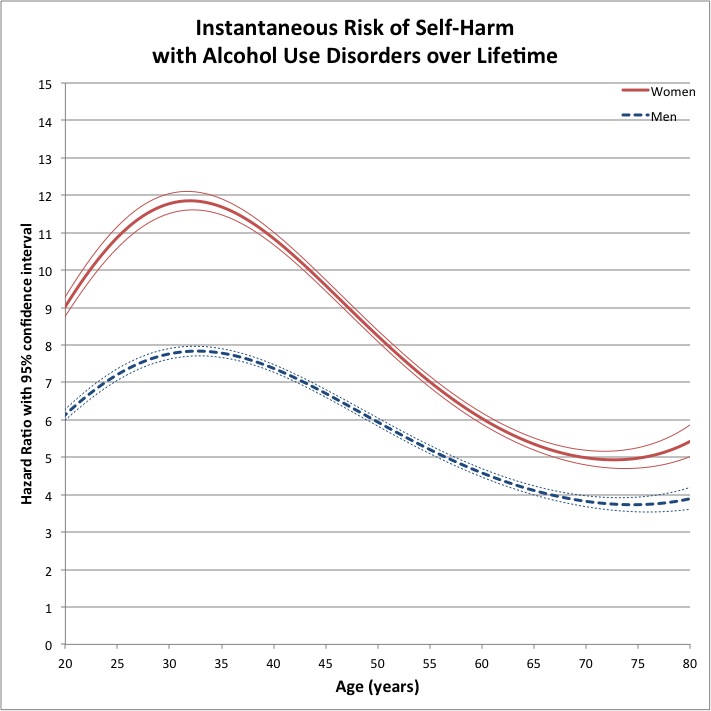


# Figure S21. Instantaneous Risk of Other Violent Injury with Alcoholic Use Disorders over the lifetime (French National Hospital Discharge database 2008-2012)


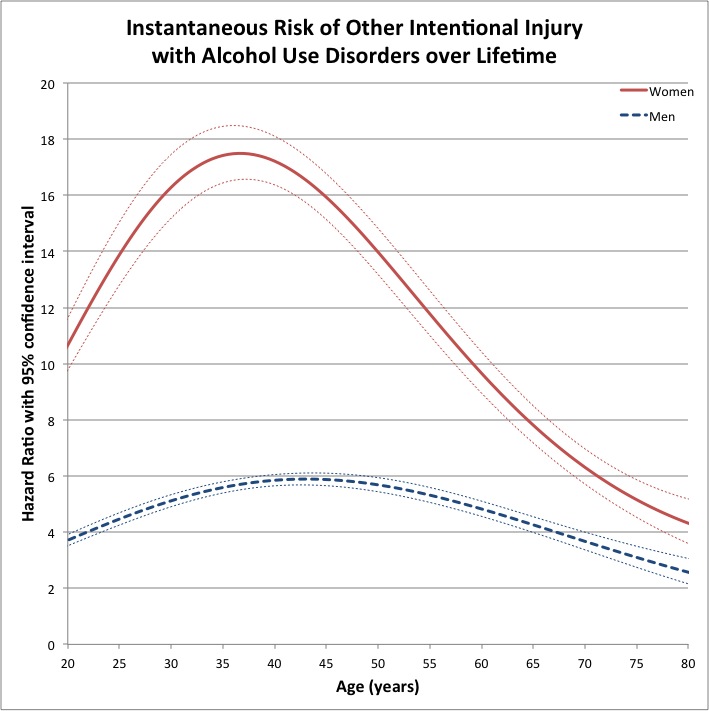


Reference List

1. Rehm J, Mathers C, Popova S, Thavorncharoensap M, Teerawattananon Y, Patra J. Alcohol and Global Health 1 Global burden of disease and injury and economic cost attributable to alcohol use and alcohol-use disorders. Lancet. 2009; 373:2223-2233.

2. Quan H, Sundararajan V, Halfon P, Fong A, Burnand B, Luthi JC *et al*. Coding algorithms for defining comorbidities in ICD-9-CM and ICD-10 administrative data. Medical care. 2005; 43:1130-1139.

3. International Agency for Research on Cancer. GLOBOCAN 2012: Estimated Cancer Incidence, Mortality and Prevalence Worldwide in 2012. Lyon, France: World Health Organization, International Agency for Research on Cancer; 2016.

4. Lozano R, Naghavi M, Foreman K, Lim S, Shibuya K, Aboyans V *et al*. Global and regional mortality from 235 causes of death for 20 age groups in 1990 and 2010: a systematic analysis for the Global Burden of Disease Study 2010. Lancet. 2012; 380:2095-2128.
